# Susceptibility of *Mycobacterium immunogenum* and *Pseudomonas fluorescens* to Formaldehyde and Non-Formaldehyde Biocides in Semi-Synthetic Metalworking Fluids

**DOI:** 10.3390/ijms12010725

**Published:** 2011-01-20

**Authors:** Suresh B. Selvaraju, Izhar U. H. Khan, Jagjit S. Yadav

**Affiliations:** Environmental Genetics and Molecular Toxicology Division, Department of Environmental Health, University of Cincinnati Medical Center, Cincinnati, OH 45267, USA

**Keywords:** metalworking fluid, Mycobacterium immunogenum, Pseudomonas fluorescens, biocide susceptibility, biocide resistance, formaldehyde

## Abstract

*Mycobacterium immunogenum*, a newly identified member of the *Mycobacterium chelonae_M. abscessus* complex is considered a potential etiological agent for hypersensitivity pneumonitis (HP) in machine workers exposed to contaminated metalworking fluid (MWF). This study investigated the biocidal efficacy of the frequently applied commercial formaldehyde-releasing (HCHO) biocides Grotan and Bioban CS 1135 and non-HCHO type biocides Kathon 886 MW (isothiazolone) and Preventol CMK 40 (phenolic) toward this emerging mycobacterial species (*M. immunogenum*) in HP-linked MWFs, alone and in presence of a representative of the Gram-negative bacterial contaminants, *Pseudomonas fluorescens*, using two semi-synthetic MWF matrices (designated Fluid A and Fluid B). Relative biocide susceptibility analysis indicated *M immunogenum* to be comparatively more resistant (2–1600 fold) than *P. fluorescens* to the tested biocides under the varied test conditions. In terms of minimum inhibitory concentration, Kathon was the most effective biocide against *M. immunogenum*. Fluid factors had a major effect on the biocide susceptibility. Fluid A formulation provided greater protective advantage to the test organisms than Fluid B. Fluid dialysis (Fluid A) led to an increased biocidal efficacy of Grotan, Kathon and Preventol against *M. immunogenum* further implying the role of native fluid components. Used fluid matrix, in general, increased the resistance of the two test organisms against the biocides, with certain exceptions. *M. immunogenum* resistance increased in presence of the co-contaminant *P. fluorescens*. Collectively, the results show a multifactorial nature of the biocide susceptibility of MWF-colonizing mycobacteria and highlight the importance of more rigorous efficacy testing and validation of biocides prior to and during their application in metalworking fluid operations.

## 1. Introduction

Modern water-based metalworking fluids (MWFs) used as coolants and lubricants in many metalworking industries are available as synthetic or semi-synthetic formulations. These fluids are vulnerable to microbial colonization, the extent of which may depend on the fluid composition. Microbial growth reduces the quality and effectiveness of these fluids and may cause occupational health hazards in the exposed machinists [1–4]. Dilution of the commercially available MWF concentrate to a desired concentration with tap water before application provides an environment favorable for microbial contamination and growth. Furthermore, in-use metalworking fluids are well aerated through agitation and maintained at a temperature favorable for microbial growth in an open system, which collectively enhance the establishment of microbial consortia in MWF. Biocide application is a common practice to control the microbial build-up in metalworking fluids. However, it is generally believed that initial appearance of Gram-negative bacteria which degrade MWF characteristics prompts repeated biocide applications, which in turn select for the relatively biocide resistant microbial groups particularly mycobacteria.

Several studies have suggested a possible relationship between MWF microflora and respiratory health hazards in metal workers particularly hypersensitivity pneumonitis (HP) [2,3,5–7]. Initial studies indicated the role of Gram-negative bacteria particularly pseudomonads [8,9] and their cell wall lipopolysaccharide (endotoxin) in causing occupational respiratory illnesses via exposure to the contaminated MWFs or their aerosols [10,11]. However, the attention soon focused on prevalence of the mycobacteria species of the *Mycobacterium chelonae_M. abscessus* complex in fluids associated with occupational respiratory illnesses [2,3,7,12–17]. In particular, *M. immunogenum* has been reported as the predominant or one of the predominant species colonizing MWF [18–23] and considered as a possible etiological agent for occupational hypersensitivity pneumonitis [6,15,18].

Limited controlled studies have been undertaken on evaluation of the effect of commercially available biocides on specific microorganisms of occupational health significance in industrial MWF formulations and effect of various fluid factors affecting their biocide efficacy [24–27]. Recently we reported efficacy of selected MWF biocides in a synthetic MWF formulation and saline [24]. Here we report the potential biocidal activities of formaldehyde-releasing (HCHO) and non-HCHO type biocides in commercial semi-synthetic metalworking fluid formulations toward *M. immunogenum*, an isolate from metalworking fluids associated with occupational HP, and *P. fluorescens*, a representative of the Gram-negative organisms in these fluids. These test organisms were studied individually and in mixed suspensions with an aim to determine the effect of fluid types, fluid dialysis, fluid use, and microbial mixture on biocide efficacy in MWF matrix. To our knowledge this is the first comprehensive biocide evaluation study in commercial semi-synthetic MWF formulations against the MWF-isolated *Mycobacterium* species alone and in presence of a common Gram-negative contaminant.

## 2. Results and Discussion

### 2.1. Effect of Fluid Type on Biocide Efficacy

**Figure 1:**
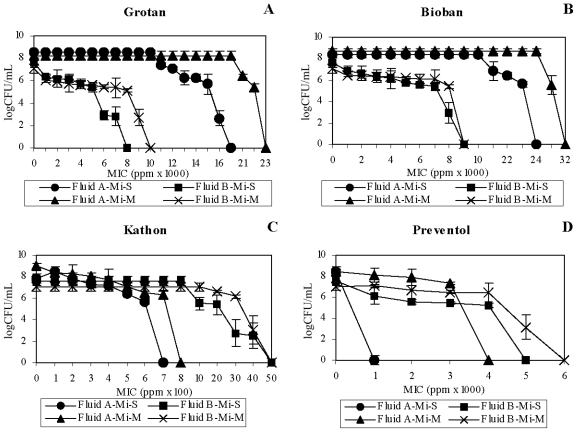
Effect of fluid type on biocidal efficacy toward *M. immunogenum*. Two different semi-synthetic fluid types (Fluid A and Fluid B) were used to investigate the biocidal activity of four individual test biocides namely, Grotan (Panel **A**), Bioban (Panel **B**), Kathon (Panel **C**) and Preventol (Panel **D**) in terms of minimum inhibitory concentration (MIC), either in single culture (pure) suspension or in mixed culture suspension (1:1 ratio with *P. fluorescens*) of *M. immunogenum*. The MIC values were based on 100% killing of the test organism and are means of the triplicates. The test organism was exposed to varying levels of the individual biocides for 60 min each in the two test MWF matrices. Abbreviations: Mi, *M. immunogenum*; S, Single culture suspension (pure suspension); M, Mixed culture suspension.

**Figure 2:**
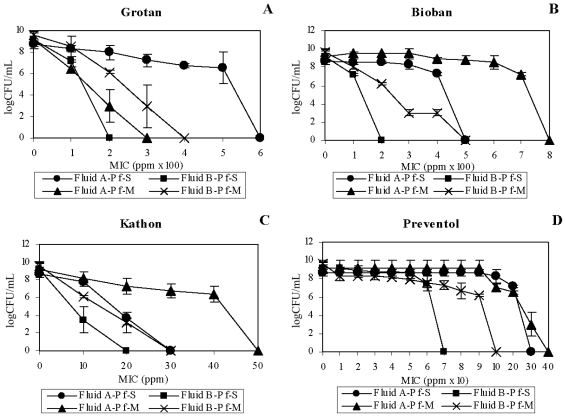
Effect of fluid type on biocidal efficacy toward *P. fluorescens*. Two different semi-synthetic fluid types (Fluid A and Fluid B) were used to investigate the biocidal activity of four individual test biocides namely, Grotan (Panel **A**), Bioban (Panel **B**), Kathon (Panel **C**) and Preventol (Panel **D**) in terms of minimum inhibitory concentration (MIC), either in single culture (pure) suspension or in mixed culture suspension (1:1 ratio with *M. immunogenum*) of P*. fluorescens*. The MIC values were based on 100% killing of the test organism and are means of the triplicates. The test organism was exposed to varying levels of the individual biocides for 15 min each in the two test fluid matrices. Abbreviations: Pf, *P. fluorescens*; S, Single culture suspension (pure suspension); M, Mixed culture suspension.

The test organisms, *M. immunogenum* and *P. fluorescens*, showed increased estimated MIC of the HCHO-releasing biocides (2.7–3.6 fold) in Fluid A as compared to Fluid B matrix (Figure 1A, B). The data indicate an additive effect of the native MWF components of Fluid B and the HCHO-biocides. A similar trend of increased MIC of *P. fluorescens* in Fluid A was observed against Grotan (3 fold; Figure 2A) and Bioban (1.6–2.5 fold; Figure 2B). However, *P. fluorescens* in mixed suspension showed a reverse trend with higher Grotan MIC estimate (1.3 fold) in Fluid B compared to Fluid A (Figure 2A).

In contrast to the HCHO-biocides, Fluid A enhanced the microbicidal activity of non-HCHO biocides (Kathon and Preventol) against *M. immunogenum* (Table 1). This was also apparent from the increased MIC estimates (1.5–7 fold) in Fluid B compared to Fluid A matrix (Figure 1C, D). However, the trend was opposite for *P. fluorescens*, where an increased MIC estimate (1.5–4.3 fold) in Fluid A was observed with non-HCHO biocides (Figure 2C, D).

Taken together, our results revealed a differential biocidal activity of HCHO- and non-HCHO biocides in the two fluid formulations (Fluid A *versus* Fluid B). For *M. immunogenum*, HCHO-biocides (Grotan and Bioban) were effective at lower concentrations in Fluid B as compared to Fluid A. On the other hand, non-HCHO biocides (Kathon and Preventol) were effective in Fluid A at lower concentrations. This could be because of a role of compatibility between the biocide and the fluid composition. In this context, earlier studies had suggested that interaction between corrosion inhibitors (ingredients in MWF) and added biocides was crucial in the biocidal efficacy outcome [1,28]. Considering this, presence of amines and borates in Fluid B might have exacerbated the biocidal activity of HCHO-biocides in this fluid as compared to Fluid A. A reverse trend observed with the non-HCHO biocides (more effective in Fluid A than in Fluid B) suggested the importance of the biocide type in this compatibility equation. In addition, Isothiazolone (Kathon) biocides are inactivated by nucleophiles [29]. This may explain the decreased biocidal activity of Kathon in Fluid B formulation, which is rich in amines (15%), as compared to Fluid A. In contrast with mycobacteria, *P. fluorescens* showed a different pattern of biocidal activity as all four biocides (both HCHO- and non-HCHO) were in general effective at lower concentrations in the Fluid B matrix as compared to Fluid A. This indicates the role of organism type as an additional factor in the biocide-MWF interaction phenomenon in determining the overall biocidal efficacy. In other words, the biocidal activity depends on the interaction among the active ingredient of the biocide, the native inhibitory components of the fluid, and the target organism.

### 2.2. Effect of Fluid Dialysis on Biocidal Activity

Pristine (unused) Fluid A was selected to study the effect of dialysis on the efficacy of the test biocides. The dialysis step was meant to investigate the role of fluid constituents less than 3500 molecular weight size in biocide performance.

**Figure 3:**
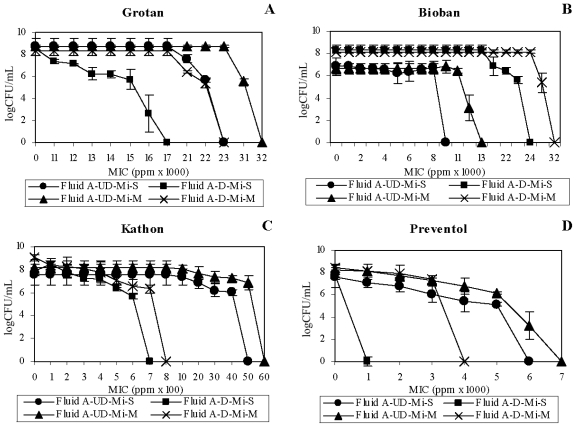
Effect of fluid dialysis on biocidal efficacy toward *M. immunogenum*. Undialyzed and dialyzed forms of Fluid A were used to compare the biocidal activity of four individual test biocides namely, Grotan (Panel **A**), Bioban (Panel **B**), Kathon (Panel **C**) and Preventol (Panel **D**) in terms of minimum inhibitory concentration (MIC), either in single culture (pure) suspension or in mixed culture suspension (1:1 ratio with *P. fluorescens*) of *M. immunogenum*. The MIC values were based on 100% killing of the test organism and are means of the triplicates. The test organism was exposed to varying levels of the individual biocides for 60 min each in the two test MWF matrices. Abbreviations: Mi, *M. immunogenum*; S, Single culture suspension (pure suspension); M, Mixed culture suspension; UD, Undialyzed; D, Dialyzed. Fluid dialysis was meant to remove the native inhibitory substances.

**Figure 4:**
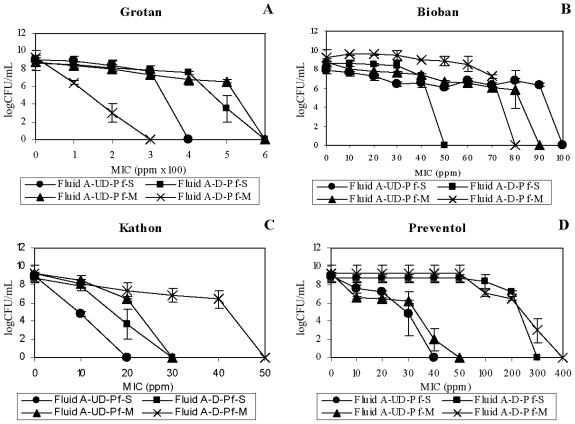
Effect of fluid dialysis on biocidal efficacy toward *P. fluorescens*. Two forms of Fluid A (undialyzed and dialyzed) were used to compare the biocidal activities of Grotan (Panel **A**), Bioban (Panel **B**), Kathon (Panel **C**) or Preventol (Panel **D**) toward *P. fluorescens*. The activities were compared in terms of mean minimum inhibitory concentration (MIC) in the given fluid matrix simulated with either a single culture (pure) suspension or mixed culture suspension (1:1 ratio with *M. immunogenum*) of *P. fluorescens*. The test organism was exposed to the varying levels of biocides for 15 min each in the two MWF matrices. Fluid dialysis was meant to remove the native inhibitory substances. Abbreviations: Pf, *P. fluorescens*; S, Single culture suspension (pure suspension); M, Mixed culture suspension; UD, Undialyzed; D, Dialyzed.

For HCHO-biocides, *M. immunogenum* showed a mixed trend on the effect of fluid dialysis on the biocide efficacy either singly or in mixed suspension. For instance, Grotan showed increased MIC estimates (Figure 3A) in undialyzed fluid (23,000 ppm–32,000 ppm) compared to those after dialysis (17,000 ppm and 23,000 ppm), whereas Bioban showed increased MIC estimates in dialyzed fluid (Figure 3B). In contrast, *P. fluorescens* showed an increased MIC estimate (1.1–2 fold) for both the HCHO-biocides in the undialyzed fluid compared to dialyzed matrix (Figure 4A, B); the exception being the single culture suspension of *P. fluorescens* against Grotan where increased MIC estimate was observed (Figure 4A).

In contrast with the HCHO-biocides, the non-HCHO biocides showed an increased MIC estimate (1.75–7.5 fold) against *M. immunogenum* in undialyzed fluid compared to dialyzed fluid (Figure 3C, 4C). *P. fluorescens* showed an opposite trend with increased MIC estimates (1.5–8 fold) in dialyzed fluid (Figure 3D, 4D). Collectively, the results on non-HCHO biocides showed that dialysis caused an increase in biocide sensitivity of *M. immunogenum*, unlike *P. fluorescens* which showed decrease in sensitivity due to dialysis.

Taken together, the fluid dialysis pre-step did seem to increase the biocide susceptibility of *M. immunogenum* against Grotan, Kathon and Preventol. This trend might be either because of removal of certain native fluid components potentially showing antagonism toward the biocide or due to a protective effect of <3500 molecular weight (M.W.) size components of the fluid that got removed by the dialysis process.

On the other hand, a protective effect of the dialyzed fluid toward non-HCHO biocides (Kathon and Preventol) was observed for *P. fluorescens*. This protection may be due to removal of certain native components (<3500 M.W.) inhibitory to this organism. In contrast, HCHO-biocides showed increased biocidal activity in dialyzed as compared to undialyzed matrix, showing a possible protective effect of the native components toward the test organism. In an earlier study, specific MWF components including borate esters, triethanolamine, dicarboxylic acid, phosphate ester and polyglycol were shown to serve as sole carbon and/or nitrogen sources for the bacterial isolates and were indicated to be possibly responsible for the observed protective effect toward biocide action [30]. Grotan activity against pure culture suspension was the exception. Reduction in pH [28] could be the possible reason for this decreased activity of Grotan in dialyzed fluid, as this biocide requires slightly higher pH range (around 8.5) for its normal HCHO release and activity [28].

### 2.3. Effect of Fluid Usage on Biocidal Activity

**Figure 5:**
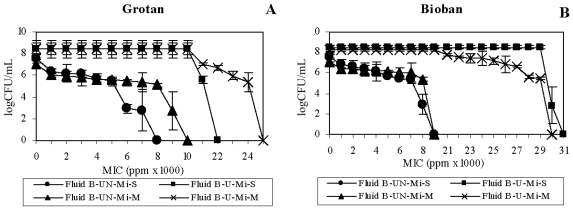

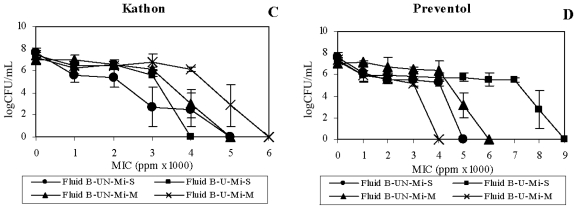
Effect of fluid use on biocidal efficacy toward *M. immunogenum*. Pristine (unused) and used Fluid B matrices were used to compare the biocidal efficacy of the four test biocides, Grotan (Panel **A**), Bioban (Panel **B**), Kathon (Panel **C**) and Preventol (Panel **D**), against *M. immunogenum* to know the effect of fluid use on biocidal activity. *M. immunogenum* was exposed to the varying concentrations of each test biocide as single culture (pure) suspension or mixed suspension (1:1 ratio with *P. fluorescens*) for 60 min each in the two MWF matrices. Abbreviations: Mi, *M. immunogenum*; S, Single culture suspension (pure suspension); M, Mixed culture suspension; UN, Unused; U, Used.

**Figure 6:**
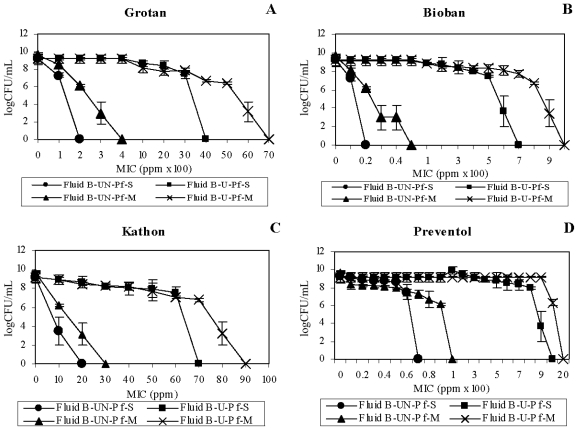
Effect of fluid use on biocidal efficacy toward *P. fluorescens*. Pristine (unused) and used Fluid B matrices were used to compare the biocidal efficacy of the four test biocides, Grotan (Panel **A**), Bioban (Panel **B**), Kathon (Panel **C**) and Preventol (Panel **D**), against *P. fluorescens* to know the effect of fluid use on biocidal activity. *P. fluorescens* was exposed to the varying concentrations of each test biocide as single culture (pure) suspension or mixed suspension (1:1 ratio with *M. immunogenum*) for 15 min each in the two MWF matrices. Abbreviations: Pf, *P. fluorescens*; S, Single culture suspension (pure suspension); M, Mixed culture suspension; UN, Unused; U, Used.

Both the HCHO-biocides were less effective in the used fluid matrix than unused (pristine) fluid matrix against the two test organisms (*M. immunogenum* and *P. fluorescens*) in either (single or mixed) culture suspension. A 2.5–3.4 fold increase in MIC estimate was observed in the used fluid compared to unused fluid when the two HCHO-biocides were tested against *M. immunogenum* (Figure 5A, B). In case of *P. fluorescens*, a much higher MIC (18–35 fold) was required to kill the organisms in the used fluid (Figure 6A, B).

Interestingly, the efficacy of the non-HCHO biocides toward *M. immunogenum* showed a mixed trend in the used *versus* unused matrix. Kathon efficacy toward the single culture (pure) suspension was higher in the used fluid matrix (MIC value of 4000 ppm) when compared to the unused matrix (MIC value of 5000 ppm; Figure 5C). The opposite was observed with mixed culture suspension of *M. immunogenum* where, the biocidal efficacy was higher in the unused fluid (MIC value of 5000 ppm) as compared to the used fluid (MIC value of 6000 ppm; Figure 5C). With Preventol, the efficacy toward *M. immunogenum* (Figure 5D) in single culture (pure) suspension was higher in the unused fluid matrix (refined MICs of 5000 ppm in pristine *versus* 9000 ppm in used) whereas a reverse pattern was true for the mixed culture suspension (refined MICs of 6000 ppm in unused *versus* 4000 ppm in used).

Unlike *M. immunogenum*, *P. fluorescens* showed a clear trend of increased MIC estimate (3–20 fold) for non-HCHO biocides in used fluid compared to unused fluid matrix (Figure 6C, D). This showed a differential effect of the used matrix for the two test organisms, being protective for pure suspension type for *M. immunogenum* and for both suspension types for *P. fluorescens*.

Taken together, the results showed that the fluid use, in general, increased the antimicrobial resistance of the two test organisms, *M. immunogenum* and *P. fluorescens*, against the biocides, with the exceptions discussed above. The clearly detectable protective effect of the used MWF matrix over pristine matrix might possibly be because of the presence of organic debris or fluid degradation products or loss of inherent inhibitory components in the used fluid. Earlier reports suggested that production of nucleophiles (amino acids, nucleic acids), drop in pH, and formation of enzymes could cause the resistance development and in turn lead to the enhanced survival of microorganisms [28]. Potential generation of these organic metabolites (nucleophiles, enzymes) may be expected in the field setting considering the practice of open tank storage and prolonged circulation of MWF, making it a reservoir for microbial contamination and activity.

### 2.4. Increased Biocide Resistance of M. Immunogenum

*M. immunogenum* was comparatively more resistant (2–1600 fold) than *P. fluorescens* with the tested biocides under the varied test conditions as also indicated by a much higher MIC range for the former than the latter (Table 1). For *M. immunogenum*, non-HCHO biocides were effective at a lower MIC range (<10,000 ppm) than the HCHO-biocides (>10,000 ppm) with some exceptions. Likewise, for *P. fluorescens*, the non-HCHO biocides required lower doses (MICs < 100 ppm) as compared to the HCHO- (>100 ppm) biocides (Table 1). More biocide resistance in *M. immunogenum* than *P. fluorescens* against all test biocides might be due to its intrinsic resistance mechanism (lipid-rich cell wall, efflux pumps, *etc.*) against chemical biocides. Overall, the results of this study indicated that the non-HCHO biocides (Kathon and Preventol) are more efficient in controlling the test organisms, *M. immunogenum* and *P. fluorescens*, compared to the HCHO-releasing biocides (Grotan and Bioban). Between the two non-HCHO biocides, Kathon (an Isothiazolone biocide) was the better performing biocide than Preventol (a phenolic biocide). In contrast, no such clear difference was observed between the efficacy of the two HCHO-releasing biocides (Grotan and Bioban). Although Kathon appeared to be the most effective among the four tested commercial biocides against Mycobacterium, there was some degree of Kathon resistance observed in this organism as compared to Pseudomonas. Isothiozolone (the active component in Kathon) is known to be inactivated by thiols. Mycobacteria produce mycothiol, a unique thiol compound comprised of *N*-acetylcysteine amide [15]. The mycobacterial thiol may detoxify the thiol reactive active component in Kathon biocide [31] thereby reducing its efficacy. Since the Isothiazolone has been reported to be activated in alkaline pH [1], the prevalence of high pH in MWF matrix (pH 8–10) might have caused an increased biocidal activity of Kathon in MWF. Preventol, a phenol-based biocide exhibits surface-active properties and thus causes generalized disruption of the cell membrane, which results in cell lysis and progressive loss of intracellular contents of organisms [32]. However, such phenolic biocides are weak in physical interaction with lipid-rich components of the bacterial cell wall and this phenomenon could be responsible for the relatively greater resistance of *M. immunogenum* to Preventol [33].

The relatively lower effectiveness (in terms of MIC) of HCHO-releasing biocides, Grotan and Bioban, against *M. immunogenum* could be partially attributed to their mode of action, which involves surface activity of the released active component (formaldehyde) on bacterial cells [34]. The hydrophobic and waxy nature of mycobacterial cell wall discourages the interaction of formaldehyde and thus provides protection against HCHO-biocides. The mycothiol provides another important protective mechanism against HCHO-biocides via detoxification of the active component formaldehyde [31].

### 2.5. Role of Co-Contamination in Biocide Susceptibility of the Test Organisms

In general, both the test organisms showed increased biocide resistance (0–4 fold) in mixed culture suspensions compared to single culture (pure) suspensions except in a few instances. *M. immunogenum* was found to be more resistant in mixed suspension than in pure suspension, to both HCHO- and non-HCHO biocides in all matrices; the exceptions being Bioban and Preventol in used Fluid B matrix. The relative biocidal resistance of *P. fluorescens* in mixed suspension toward different test biocides in all matrices was also increased, except for Grotan in dialyzed Fluid A. An increased biocidal resistance of both *M. immunogenum* and *P. fluorescens* in presence of a co-contaminant suggests a possible protective mechanism among the microbial communities in MWF environment leading to their enhanced ability to resist biocide. The extent of protection varied with the biocide possibly due to differential distribution of the individual biocides between the two organisms and a consequent dilution effect. Reasons for the exceptions (Bioban and Preventol for *M. immunogenum* and Grotan for *P. fluorescens*) are however not clear.

## 3. Experimental Section

### 3.1. Microbial Isolates and Culture Conditions

Two test organisms, namely, *Mycobacterium immunogenum* (ATCC 700506), an MWF-isolate, and *Pseudomonas fluorescens* (ATCC 13525), a representative of the Gram-negative organisms in MWF, were used in this study. The organisms were cultured using M7H9 broth and M7H10 agar (Difco, Detroit, MI) supplemented with Oleic acid-Albumin-Dextrose-Catalase enrichment (OADC, BD Biosciences, Sparks, MD). The cultures of *M. immunogenum* and *P. fluorescens* were grown in 40 mL of broth with continuous shaking (150 rpm) at 37 °C for 120 h and at 25 °C for 24 h, respectively.

### 3.2. Metalworking Fluids

Two commercial semi-synthetic metalworking fluid formulations, arbitrarily designated for proprietary reasons as Fluid A (pristine) and Fluid B (pristine or in-use), were obtained from an industrial setting for use in this study. According to the manufacturer’s specifications, Fluid A is a “biocide free” formulation whereas Fluid B is a “biocide added” formulation and the two have some basic compositional differences as well. Initially, we used a 5% (v/v) dilution of these MWF formulations but no bacterial growth was observed in either of the matrices indicating the presence of certain inhibitory constituents in these formulations. This warranted the selection and use of a noninhibitory fluid concentration (2% v/v), standardized in our hands for the purpose, for the two test organisms (data not shown). A field used version of Fluid B obtained from the same commercial source that supplied the pristine fluid was used for comparison in this study.

### 3.3. Biocides

Four commercial biocides were evaluated in this study. Of these, two biocides were HCHO-releasing type, namely Grotan (Troy Chemical Corp., Newark, NJ) and Bioban CS 1135 (DOW Chemical Co., Midland, MI). The active ingredients were hexahydro-1,3,5-tris(2- hydroxyethyl)-s-triazine (78.9%) for Grotan and 4,4-Dimethyloxazolidine-3,4,4-trimethyloxazolidine (76%) for Bioban. An isothiazolone group biocide, Kathon 886 MW (Rohm & Hass Co., Philadelphia, PA) with 14.1% 5-chloro-2-methyl-4-isothiazolin-3-one as the active ingredient and a phenolic biocide, Preventol CMK 40 (Bayer Chemicals Corp., Pittsburgh, PA) with 4-chloro-3-methylphenol sodium salt (46.13%) as the active ingredient, were also tested.

### 3.4. Fluid Dialysis

Dialysis strategy (using 3500 M.W. cut-off membrane tubing) was used to get rid of the native inhibitory components in Fluid A matrix and to remove the blended biocide as well as native inhibitory components in Fluid B matrix. To study the effect of fluid dialysis process on inhibitory potential of a test biocide, 2%-undialyzed and 2%-dialyzed forms of pristine Fluid A were compared. To study the effect of fluid usage, dialyzed version of the pristine and used Fluid B matrices were compared.

### 3.5. Biocide Susceptibility Testing

Same batch of the culture for a given test organism was used in all treatments with a given test biocide to minimize the experimental variations. The bacterial cultures were grown in 40 mL of Middlebrook (MB) broth at corresponding temperature-time conditions to a cell density equivalent to 120 Klett reading measured using Klett Photoelectric Colorimeter (Klett, New York). The cell counts corresponding to this Klett reading were determined to estimate the dilution requirements for achieving a constant initial number of cells (10^8^ CFU/mL) in MWF matrix for the biocide efficacy experiments. To understand the effect of co-contaminantion on biocide sensitivity of individual test organisms, the test fluid was spiked with the test organisms, individually or in mixture (1:1 ratio), and the minimum inhibitory concentration (MIC), in terms of concentration needed to kill all cells, was determined for individual biocides.

The test organisms were treated with the individual biocides singly and in mixed suspension at different biocide concentrations in a two-step experiment. In the first step, the treatment involved a random broad concentration range of the biocide (100, 1000, 10,000 and 100,000 ppm) for different contact times (15, 30, 45 and 60 min) to determine the MIC estimates. In the subsequent step, a refined MIC value was determined by varying the concentrations between the estimated MIC concentration and its nearest lower concentration, at the estimated contact time. For instance, if the MIC estimate observed in the first step was 10,000 ppm, then we tested 1000 to 10,000 ppm range using 1000 ppm increments in the second step. In case of *M. immunogenum*, a further refinement of the MIC value was achieved by an additional narrowing down step.

Viability of the test suspensions, after biocide treatment and serial dilution in neutral phosphate buffered saline (PBS), was estimated by plating on the M7H10 agar followed by incubation at 37 °C for 120 h (*M. immunogenum*) and 25 °C for 24 h (*P. fluorescens*) as described earlier (24). In case of mixed suspension treatments, selective counts for the individual test organisms were obtained by differentially incubating the two different sets of M7H10 agar plates at either temperature of incubation (37 °C for *M. immunogenum* and 25 °C for *P. fluorescens*), as above.

## 4. Conclusions

*M. immunogenum* was relatively more resistant to the test biocides as compared to the Gram-negative test organism *P. fluorescens*. In addition, higher amounts of the HCHO-releasing biocides (Grotan and Bioban) were needed as compared to the non-HCHO biocides (Kathon and Preventol) to completely inactivate *M. immunogenum* in semi-synthetic MWF matrix. The observations pointed to the limitation of these existing commonly applied commercial MWF biocides in terms of their efficacy to control mycobacteria in these fluids. Hence, in addition to the need for development and evaluation of novel biocides, screening of a library of existing non-HCHO biocides against MWF-relevant test strains may be pursued to identify potentially more effective biocides for MWF-colonizing mycobacteria. On the other hand, the study highlights the role of fluid factors in understanding the efficacy of biocides toward the test organisms in metalworking fluids. The results showed a fluid type-dependent differential biocidal efficacy, indicating the importance of compatibility between the biocide and MWF matrix. Fluid dialysis studies indicated varying interaction of the inherent components of MWF with individual biocide groups thereby affecting the biocide efficacies. Used fluid matrix conferred increased resistance to the test organisms. This implies that prior fluid colonization activities including microbial degradation of MWF and biocide and further metabolic activities of microbes on the degraded products may lead to enhanced survival and growth of microbial contaminants and poor longevity of a biocide. The observed mutual protection against biocide killing between *P. fluorescens* and *M. immunogenum* in a mixture indicates that once the microbial communities containing Gram-negative and acid-fast bacteria are developed in the metalworking fluid environment they are able to tolerate relatively higher concentrations of biocides possibly due to biocide distribution and dilution effect. The above observations on multifactorial nature of the biocide susceptibility of mycobacteria in MWF suggest that efficient biocide control of microorganisms in these fluids would require proper fluid management practices including regular monitoring of the critical fluid factors in an industrial setting.

## Figures and Tables

**Table 1:** MIC estimates for different biocides in MWF based on complete growth inhibition at a particular contact time.

Biocide	Matrices	Biocide Concentration (ppm) and Growth Inhibition															
		*M. immunogenum* (60 min)								*P. fluorescens* (15 min)							
		100		1000		10,000		100,000		100		1000		10,000		100,000	
		S	M	S	M	S	M	S	M	S	M	S	M	S	M	S	M
Grotan	Fluid A(NUD)	+	+	+	+	+	+	−	−	+	+	−	−	−	−	−	−
	Fluid A(ND)	+	+	+	+	+	+	−	−	+	+	−	−	−	−	−	−
	Fluid B(ND)	+	+	+	+	−	−	−	−	+	+	−	−	−	−	−	−
	Fluid B(UD)	+	+	+	+	+	+	−	−	+	+	+	+	−	−	−	−
Bioban	Fluid A(NUD)	+	+	+	+	−	+	−	−	−	−	−	−	−	−	−	−
	Fluid A(ND)	+	+	+	+	+	+	−	−	−	−	−	−	−	−	−	−
	Fluid B(ND)	+	+	+	+	−	−	−	−	−	−	−	−	−	−	−	−
	Fluid B(UD)	+	+	+	+	+	+	−	−	+	+	−	−	−	−	−	−
Kathon	Fluid A(NUD)	+	+	+	+	−	−	−	−	−	−	−	−	−	−	−	−
	Fluid A(ND)	+	+	−	−	−	−	−	−	−	−	−	−	−	−	−	−
	Fluid B(ND)	+	+	+	+	−	−	−	−	−	−	−	−	−	−	−	−
	Fluid B(UD)	+	+	+	+	−	−	−	−	−	−	−	−	−	−	−	−
Preventol	Fluid A(NUD)	+	+	+	+	−	−	−	−	−	−	−	−	−	−	−	−
	Fluid A(ND)	+	+	−	+	−	−	−	−	+	+	−	−	−	−	−	−
	Fluid B(ND)	+	+	+	+	−	−	−	−	−	−	−	−	−	−	−	−
	Fluid B(UD)	+	+	+	+	−	−	−	−	+	+	−	+	−	−	−	−

S: Single culture suspension; M: Mixed culture suspension;+: Growth on the plates;−: No growth; NUD: New undialyzed fluid; ND: New dialyzed fluid; UD: Used dialyzed fluid.

## Abbreviations

HCHO-biocide: formaldehyde-releasing biocide
ai: active ingredient
MWF: metalworking fluid
MIC: minimum inhibitory concentration
